# Predictive Power of a Radiomic Signature Based on ^18^F-FDG PET/CT Images for EGFR Mutational Status in NSCLC

**DOI:** 10.3389/fonc.2019.01062

**Published:** 2019-10-15

**Authors:** Xiaofeng Li, Guotao Yin, Yufan Zhang, Dong Dai, Jianjing Liu, Peihe Chen, Lei Zhu, Wenjuan Ma, Wengui Xu

**Affiliations:** ^1^Department of Molecular Imaging and Nuclear Medicine, Tianjin Medical University Cancer Institute and Hospital, Tianjin, China; ^2^National Clinical Research Center for Cancer, Tianjin, China; ^3^Key Laboratory of Cancer Prevention and Therapy, Tianjin, China; ^4^Tianjin's Clinical Research Center for Cancer, Tianjin, China; ^5^Department of Breast Imaging, Tianjin Medical University Cancer Institute and Hospital, Tianjin, China

**Keywords:** epidermal growth factor receptor mutation, ^18^F-FDG PET/CT imaging, non-small cell lung cancer, prediction, radiomics

## Abstract

Radiomics has become an area of interest for tumor characterization in ^18^F-Fluorodeoxyglucose positron emission tomography/computed tomography (^18^F-FDG PET/CT) imaging. The aim of the present study was to demonstrate how imaging phenotypes was connected to somatic mutations through an integrated analysis of 115 non-small cell lung cancer (NSCLC) patients with somatic mutation testings and engineered computed PET/CT image analytics. A total of 38 radiomic features quantifying tumor morphological, grayscale statistic, and texture features were extracted from the segmented entire-tumor region of interest (ROI) of the primary PET/CT images. The ensembles for boosting machine learning scheme were employed for classification, and the least absolute shrink age and selection operator (LASSO) method was used to select the most predictive radiomic features for the classifiers. A radiomic signature based on both PET and CT radiomic features outperformed individual radiomic features, the PET or CT radiomic signature, and the conventional PET parameters including the maximum standardized uptake value (SUVmax), SUVmean, SUVpeak, metabolic tumor volume (MTV), and total lesion glycolysis (TLG), in discriminating between mutant-type of epidermal growth factor receptor (EGFR) and wild-type of EGFR- cases with an AUC of 0.805, an accuracy of 80.798%, a sensitivity of 0.826 and a specificity of 0.783. Consistently, a combined radiomic signature with clinical factors exhibited a further improved performance in EGFR mutation differentiation in NSCLC. In conclusion, tumor imaging phenotypes that are driven by somatic mutations may be predicted by radiomics based on PET/CT images.

## Introduction

Lung cancer is one of the most frequently diagnosed malignancies worldwide, and is the leading cause of cancer-related death, with a 5-year survival rate of only 15% (1). Non-small cell lung cancer (NSCLC) accounts for more than 80% of all primary lung cancers (2). From a genetic perspective, NSCLC is significantly driven by somatic mutations in some critical oncogenes, such as epidermal growth factor receptor (EGFR) (3). Subsequently, several EGFR tyrosine kinase inhibitors (TKIs) have been developed as small molecule targeted therapeutic agents for the treatment of NSCLC (4–6). However, only some groups of patients harboring an EGFR mutation have benefited from EGFR TKI therapy, even with a high percentage of EGFR expression in NSCLC (7, 8). Given the predictive role of EGFR mutational status in the efficacy of EGFR-TKI treatment, identification of EGFR mutational status in advance is crucial for selecting the most effective therapeutic strategy to achieve precise medicine (9). Currently, the assessments for EGFR mutational status are based on biopsies of tumor tissue or surgical resection acquisition (10, 11). Therefore, molecular testing to identify the mutational status may be limited by invasive procedure, long processing time, tissue sample availability and sampling error due to tumor heterogeneity. Thus, a non-invasive, direct radiographic method for the early detection of EGFR mutational status is needed.

As a functional imaging modality, non-invasive ^18^F-Fluorodeoxyglucose positron emission tomography/computed tomography (^18^F-FDG PET/CT) is widely used for the diagnosis and staging of oncology, playing an increasingly important role in the evaluation and management of cancer (12). Concurrently, ^18^F-FDG PET/CT imaging has been suggested as a part of the standard initial regimen for NSCLC patients (13). Different metabolic phenotypes captured in ^18^F-FDG PET/CT images represent different glucose metabolism styles associated with somatic mutation (14, 15). As previously reported, active mutations in EGFR could activate relevant intracellular signaling pathways to enhance tumor glycolysis; consequently, intense ^18^F-FDG uptake manifestation in PET images was observed (15). Previous studies conducted by other groups have also demonstrated a positive correlation between the oncogene mutational status and the maximum standardized uptake value (SUVmax) in PET images (16–18). Nevertheless, there have been conflicting results (19). Even though it is a widely accepted semi-quantitative imaging parameter derived from ^18^F-FDG PET/CT, SUVmax was the main cause of the controversy in these investigation. As a single pixel value, SUVmax is not able to reflect the glucose metabolism of the whole tumor. Metabolic tumor volume (MTV), defined as the volume of tumor tissues with high glycolytic activity, and total lesion glycolysis (TLG) are increasingly being recommended as a volumetric or quantitative measurement of tumor cells to overcome the partial volume effects and statistical bias induced by the usage of SUVmax (20, 21). Apart from all of these traditional PET imaging parameters, more useful information than what can be seen with the naked eye, can be captured in standard medical images (22). In particular, an accurate quantification of the spatial relationships between image voxels is significantly helpful to describe the degree of tumor heterogeneity (23). Fortunately, radiomics, which is an advanced mathematical model to quantify the spatial relationships between image voxels, is now an emerging area of interest in medical imaging (24). The high-throughput extraction of radiomic features from medical images w allows for a quantitative assessment of tumor imaging phenotypes to achieve individualized therapy and precise medicine (25). An emerging field that is closely related to radiomics is radiogenomics, which integrates imaging and genomic data to attain biological interpretation for imaging phenotypes (25). Somatic mutations affect the ability of cells to grow in otherwise non-permissive conditions. For example, a mutation in EGFR (26, 27) and/or KRAS (28, 29) may induce increased glycolysis and promotes glucose consumption via the activation of the Akt signaling pathway, and these alterations in glucose metabolism may be captured and reflected in PET imaging. Based on the assumption that the extracted radiomic metrics from medical images are linked to the molecular profile of the tumor lesion, such as some key oncogene mutational status, an increasing number of studies aim to investigate the association between somatic mutations and radiomic features in NSCLC (30–33). However, the majority of the investigations have focused on the texture analysis in CT (34–36) and/or magnetic resonance imaging (MRI) (37); whereas few studies in relation to PET/CT radiogenomics are conducted (38–40).

In the present study, we established a radiomic signature based on PET/CT images to reveal the predicative role of PET/CT radiomics in EGFR mutation status. The radiomic signature based on PET/CT images, especially when in combination with a clinical model, is believed to be capable of predicting the EGFR mutational status as a minimally or non-invasive imaging biomarker to complement the molecular test in identification of somatic mutational status.

## Materials and Methods

### Study Design and Patient Selection

This retrospective investigation was conducted with the approval of Tianjin Medical University Cancer Hospital Institutional Ethics Committee. NSCLC patients with a single pulmonary lesion (diameter > 1 cm) who underwent somatic mutation testing and diagnostic ^18^F-FDG PET/CT imaging prior to any treatment between June 2016 to July 2017 were included in this study. Histological diagnosis of primary NSCLC was confirmed by pathological examination of pulmonary surgical resection specimen. Patients were excluded if they were pregnant, lactating or had any or had any malignancies before. In addition, A total of 25 patients with NSCLC were excluded because the radioactivity accumulated in their lesions which were mainly composed of ground glass density components was too weak to be automatically measured by PET VCAR software (GE Healthcare, USA). Written informed consent was obtained from all the patients in this study, and all of the general clinicopathological characteristics of the eligible patients included were collected and summarized in Table 1. This study was performed in compliance with the Declaration of Helsinki and the relevant ethical guidelines.

**Table 1:** Demographics and clinicopathologic characteristics of eligible NSCLC patients with results for EGFR mutation status included in this study.

	**Total**	**Mutant-type EGFR**	**Wild-type EGFR**	***P*-value**
Number	115	64	51	
Age, median (range)	63 (28–77)	62.5 (33–77)	63 (28–74)	0.588
Smoking history (yes)	36 (31.3)	15 (23.4)	21 (41.2)	**0.042**
Gender				0.347
Male	53 (46.1)	27 (42.2)	26 (51.0)	
Female	62 (53.9)	37 (57.8)	25 (49.0)	
Stage				0.968
I–II	90 (78.3)	50 (78.1)	40 (78.4)	
III–IV	25 (21.7)	14 (21.9)	11 (21.6)	
Adenocarcinoma predominant subtype				<0.001
Lepidic	12 (10.4)	9 (14.1)	3 (5.9)	
Acinar	44 (38.3)	29 (45.3)	15 (29.4)	
Papillary	9 (7.8)	4 (6.3)	5 (9.8)	
Micropapillary	7 (6.1)	5 (7.8)	2 (3.9)	
Solid	13 (11.3)	0 (0)	13 (25.5)	
Mucinous	2 (1.7)	0 (0)	2 (3.9)	
Not avaliable	28 (24.4)	17 (26.5)	11 (21.6)	
Location				0.271
Upper lobe	69 (60)	40 (62.5)	29 (56.9)	
Middle lobe	9 (7.8)	7 (10.9)	2 (3.9)	
Lower lobe	34 (29.6)	15 (23.4)	19 (37.3)	
Overlapping lesion	3 (2.6)	2 (3.2)	1 (1.9)	

*EGFR, epidermal growth factor receptor; NSCLC, non-small cell lung cancer. P value < 0.05 was considered to indicate a statistically significant difference*.

### Patients Imaging

Briefly, the appropriate patient preparation (fasting for at least 6 h) and adequate blood glucose levels (<140 mg/dL) were requested before an intravenous injection of 4 MBq/kg of ^18^F-FDG was administered to all of the included patients. Then whole body PET/CT imaging on a GE Discovery elite (GE HealthCare, Waukesha, WI, USA) was performed 60 min after the ^18^F-FDG injection. Prior to the PET scan, a low-dose CT scan (helical pitch 0.75:1, 5 mm slice thickness, 120 kV and 50–80 mAs) was acquired for anatomical correlation and attenuation correction. A PET emission scan of 2 min per bed position in a three-dimensional mode was performed to integrate with the corresponding CT images. After reconstruction via an iterative algorithm, all PET imaging data were converted into SUV units. The SUV was calculated using the formula: [region of interest activity (mCi/mL)]/[injected dose (mCi)/body weight (g)]. All PET images were reviewed in consensus by two experienced PET/CT imaging-specialized experts. The volume of interest (VOI) was determined using an isocontour threshold method based on SUV using a commercial software (PET VCAR; GE Healthcare, USA) on GE Advantage Workstation 4.6 (AW 4.6). SUVmax, SUVmean and SUVpeak were calculated automatically within the VOI. MTV was defined as the tumor volume with 18F-FDG uptake segmented above a threshold SUV of 2.5. If SUVmax of the primary tumor was lower than a threshold SUV of 2.5, we regarded the MTV of the lesion as 0. TLG was calculated by multiplying MTV by SUVmean.

### Somatic Mutation Assessment

Tissue samples submitted for mutational analysis were obtained through biopsy or surgical resection. Genomic DNA of the tumor specimen was extracted using a microdissection method based on the manufacturer's protocols. The nucleotide sequences encoding the kinase domain (exons 18–24) of EGFR were amplified via a quantitative real-time polymerase chain reaction (PCR)-based method (qPCR). The presence of an appropriate PCR product was confirmed by resolving the PCR products on a 2% agarose gel. After purification, corresponding fragments on the gel were sequenced in both sense and antisense directions using an ABI PRISM® 9700 and ABI PRISM® 310 Genetic Analyzer (Applied Biosystems, USA). The sequenced data using SeqScape (Applied Biosystems) were analyzed and compared with the archived human sequence of EGFR (GenBank accession no. NG_007726.1), to identify the mutation. Of the 115 patients who were tested for somatic mutations, 64 patients were mutant-type of EGFR, whereas 51 patients tested negatively for the EGFR mutation (wild-type of EGFR).

### Measurement and Extraction of PET/CT Based Radiomic Features

**Figure 1:**
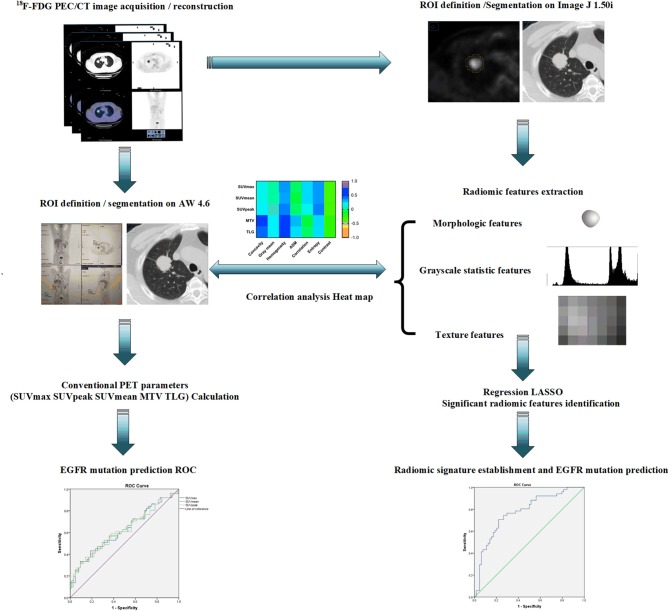
A schematic representation of a typical radiomic workflow used in this study for EGFR mutation prediction based on PET/CT images in NSCLC. EGFR, epidermal growth factor receptor; PET, positron emission tomography; CT, computed tomography; NSCLC, non-small cell lung cancer.

All segmentation was performed by two experienced PET/CT imaging-specialized experts using ImageJ 1.50i software (National Institute of Health, USA) to manually outline the contour of the region of interest (ROI) which was delineated on PET images using a 42% threshold of SUVmax. Any disagreement was resolved by consensus. All radiomic features were calculated by applying an existing automated computer program (MATLAB, The MathWorks Inc., USA). Over the segmented tumor region, a set of 38 quantitative radiomic features were extracted for each patient. The 38 features included: (1) morphological features (41, 42), such as area, perimeter, diameter, and concavity. Area was defined as the number of pixels in the tumor region; perimeter was determined by counting the number of pixels in the tumor boundary; diameter was determined by counting the maximum number of pixels between any two points; and concavity rate was defined as (A-B)/B, where A is the cross-sectional area of the tumor and B is the area of the convex hull calculated for the lesion region; (2) grayscale statistic (GSS) features (43) calculated from the histogram of the tumor voxel intensities, such as the mean, standard deviation, skewness, kurtosis, the fifth and sixth center moment, energy, and entropy; (3) texture features for quantifying intra-tumor heterogeneity calculated using the gray level co-occurrence matrix (GLCM) (44): Prior to the computation of texture features, the full intensity range of the tumor region was quantized to a smaller number of gray levels 16. For angles = 0°, 45°, 90°, and 135°, we computed four values for each of the above texture measures. Each feature was computed using a distance of one pixel. Then for each feature the mean values were used as the feature sets; Gray level-gradient co-occurrence matrix (GGCM) (45): It takes into account the information of both gray level and gradient among each pixel in an image; Gray level difference statistics (GLDS) (46): The features were calculated for displacements δ = (0, 1), (1, 1), (1, 0), (1, −1), where δ = (Δ*x*, Δ*y*), and their mean values were taken (45, 46). All the formula used to calculate GSS, GLCM, GGCM, and GLDS features were provided in Supplemental Document S1. The ensembles for boosting machine learning scheme were employed for classification, and the least absolute shrinkage and selection operator (LASSO) method (47) was used to select the most predictive features for the classifiers. LASSO is a regression analysis process utilized to identify the top-ranked or most predictive features to minimize the predicting error of outcome by altering the model fitting process. Three multivariate radiomic signatures based on PET alone, CT alone and combined PET/CT radiomic features were developed in the present study. Finally, a subset of 7 PET radiomic features and 2 CT radiomic features were finally identified and included in the PET/CT radiomic signature establishment. More importantly, several clinical factors, including age, gender, smoking status, clinical stage and lesion location, were also combined with these three developed radiomic signatures to create the corresponding integrated signatures. The typical radiomic flowchart used in this investigation is presented in Figure 1.

### Statistical Analyses

Results were expressed as the mean ± standard deviation for quantitative variables, whereas numbers and percentages were used for categorical variables. The Wilcoxon rank-sum test was used to determine whether there was a significant difference in the feature values between EGFR mutated cases and cases without the EGFR mutation. The correlations between conventional PET-derived parameters and radiomic features based on PET images were evaluated using the Spearman's coefficient r value. The predictive performance of each feature in classifying patients according to their EGFR mutation status was evaluated and quantified using the area under curve (AUC) in receiver-operating-characteristic (ROC) curve analysis. The value of AUC ranged from 0.5 to 1.0, where a value of 0.5 was interpreted with the same probability as a random guess and a value of 1.0 indicated a perfect classification. We used a Noether's test to determine whether the value of AUC was significantly greater than a random guess (AUC = 0.5). Considering the relatively small sample size included in this study, a 10-fold cross validation was utilized and repeated 10 times to calculate an average classification performance of the developed radiomic signatures. To correct for multiple comparisons, all *P*-values from the Wilcoxon rank-sum test were adjusted for multiple hypothesis testing by using the Benjamini-Hochberg procedure (48) [false-discovery rate (FDR)], with a significance threshold of 10%. Analyses were performed using R statistical software (version 3.2.2) and Statistical Package for Social Science 21.0 version (SPSS, Inc., USA).

## Results

### Comparison of Conventional PET Parameters Between EGFR Mutations

**Figure 2:**
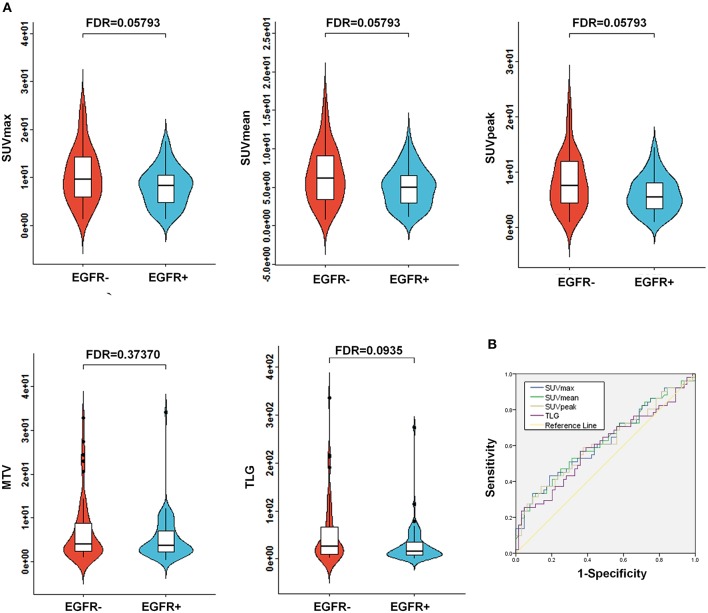
Comparison of conventional PET variables between EGFR mutated cases and wild type EGFR cases in NSCLC. **(A)** Box plots for SUVmax, SUVmean, SUVpeak, MTV, and TLG between EGFR mutations. As illustrated, significant differences were exhibited for SUVmax, SUVmean, SUVpeak, and TLG between EGFR mutations. Whereas, no marked variations were observed for MTV between the EGFR mutation positive (EGFR+) subgroup and the EGFR mutation negative (EGFR-) subgroup. **(B)** Receiver operating characteristic (ROC) curves for the prediction of EGFR mutations using the identified significant conventional PET parameters, including SUVmax, SUVmean, SUVpeak, and TLG. The area under the curve (AUC) was calculated for SUVmax, SUVmean, SUVpeak, and TLG, respectively. AUC, the area under the curve; EGFR, epidermal growth factor receptor; PET, positron emission tomography; CT, computed tomography; NSCLC, non-small cell lung cancer; SUV, standardized uptake value; MTV, metabolic tumor volume; TLG, total lesion glycolysis.

Of the 115 NSCLC patients with results for EGFR mutational status assessment included in the present study, 56% (64/115) of patients harbored a EGFR mutation (EGFR+), whereas 44% (51/115) of patients tested negatively for the EGFR mutation (EGFR-; Table 1). To assess the association between conventional PET parameters (SUVmax, SUVmean, SUVpeak, MTV, and TLG) and EGFR mutational status, we first compared the conventional PET values between the mutant-type of EGFR and wild-type of EGFR subgroups, and then conducted ROC analyses to evaluate their performances in distinguishing the EGFR mutation. For EGFR mutated NSCLC patients, the SUVmax, SUVmean, SUVpeak, and TLG were found to be underrepresented in comparison with the EGFR- subgroup, whereas no significant difference existed in the MTV between the EGFR+ and EGFR- subgroups (Figure 2A). Using ROC analyses, AUCs were assessed to evaluate the ability of the four significant conventional PET parameters to predict EGFR mutational status in NSCLC. As illustrated in Figure 2B, all three of the SUV parameters were able to significantly discriminate the mutant-type of EGFR subgroup from the wild-type of EGFR subgroup [AUC = 0.621 (*P* = 0.026), 0.624 (*P* = 0.023), and 0.615 (*P* = 0.035) for SUVmax, SUVmean, and SUVpeak, respectively], but TLG did not exhibit significant predictive power for EGFR mutation status in NSCLC [AUC = 0.597 (*P* = 0.074)].

### Correlation Between Radiomic Features Derived From PET Images and Conventional PET Parameters

**Figure 3:**
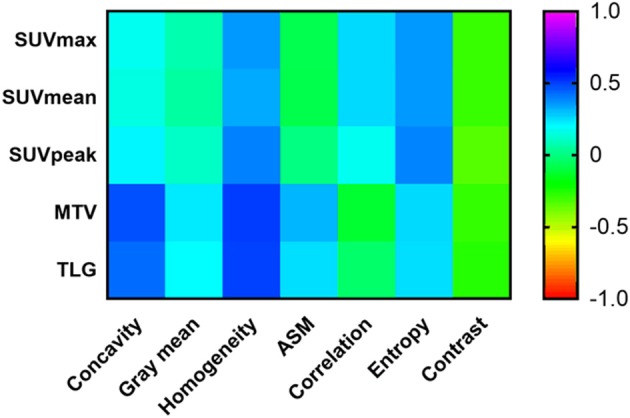
A heat map for the correlation between conventional PET parameters and the identified radiomic features extracted from PET images. PET, positron emission tomography.

In order to reduce the potential redundancy among all of the radiomic features extracted in this study, a feature selection method called LASSO was adopted to select only a subset of radiomic features to minimize the predicting error of outcome. Then the correlations between the identified radiomic features based on PET images and conventional PET parameters were further determined in the present investigation. As illustrated in Figure 3, among all of the identified PET images-derived radiomic features, homogeneity, entropy and contrast were found to be significantly correlated (*P* < 0.05) with all five of the conventional PET quantitative parameters. Spearman's coefficients between these three radiomic features and conventional parameters ranged from 0.336 to 0.500 (homogeneity), 0.252 to 0.388 (entropy), and −0.262 to −0.338 (contrast). Gray mean, concavity and ASM had poor or no significant correlations (*P* > 0.05) with SUVmax, SUVmean, and SUVpeak, whereas correlations between these three radiomic features and MTV and TLG were stronger (*P* < 0.05), with Spearman's coefficients from 0.196 to 0.474. By contrast, radiomic features called correlations were less correlated to MTV and TLG (*P* > 0.05) than to SUVmax, SUVmean, and SUVpeak (*P* < 0.05).

### Predictive Power of the PET/CT-Derived Radiomic Signature for EGFR Mutational Status

To more comprehensively evaluate the value of radiomic analysis to predict EGFR mutational status in NSCLC, we developed three radiomic signatures derived from PET images alone, CT images alone and combined PET/CT images. As shown in Table 2, among the three established radiomic signatures, the one based on PET/CT images had the most predictive power in discriminating between mutant-type of EGFR and wild-type of EGFR cases with an AUC of 0.805, an accuracy of 80.798%, a sensitivity of 0.826 and a specificity of 0.783. Meanwhile, a radiomic signature based on the identified PET radiomic features alone (AUC = 0.789) significantly outperformed a radiomics signature based on CT radiomic features alone (AUC = 0.667) in EGFR mutation discrimination in NSCLC. As several clinical parameters, such as gender, smoking status, and histopathological type, have been reported to be informative for EGFR mutational status in NSCLC (49), we combined several clinical parameters (including age, gender, smoking status, clinical stage, and lesion location) with these developed signatures to create integrated models in order to evaluate how these clinical factors affect the performance of these radiomics signatures. Consistently, combined radiomic signatures with clinical factors exhibited improved performance, especially for PET/CT radiomic signatures with an AUC of 0.822, an accuracy of 82.652%, a sensitivity of 0.821 and a specificity of 0.823 (Table 3).

**Table 2:** Radiomic signature to predict EGFR mutation in NSCLC.

**Radiomic signature**	**AUC**	**Accuracy (%)**	**Sensitivity**	**Specificity**
PET/CT	0.805	80.798	0.826	0.783
PET	0.789	79.056	0.779	0.800
CT	0.667	65.105	0.574	0.727

*EGFR, epidermal growth factor receptor; NSCLC, non-small cell lung cancer; PET, positron emission tomography; CT, computed tomography*.

**Table 3:** Radiomic signature combined with clinical models to predict EGFR mutation in NSCLC (Age, gender, smoking status, clinical stage, and lesion location were included in clinical model).

**Radiomic signature**	**AUC**	**Accuracy (%)**	**Sensitivity**	**Specificity**
PET/CT	0.822	82.652	0.821	0.823
PET	0.774	78.182	0.807	0.740
CT	0.686	68.712	0.721	0.650

*EGFR, epidermal growth factor receptor; NSCLC, non-small cell lung cancer; PET, positron emission tomography; CT, computed tomography*.

**Figure 4:**
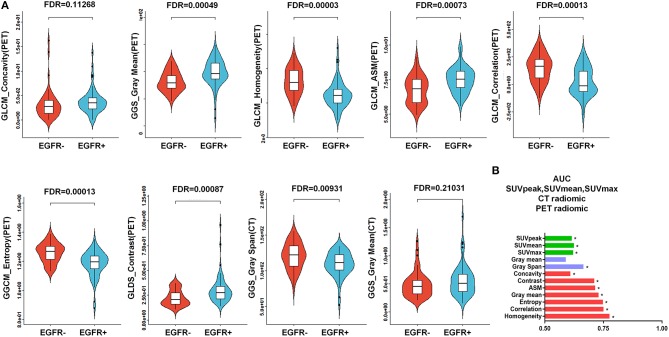
Predictive power of the PET/CT-derived radiomic signature for EGFR mutational status. **(A)** Box plots for all 7 of the identified PET-derived radiomic features and the 2 CT-based radiomic features between the mutant-type of EGFR and wild-type of EGFRsubgroups. Except for a CT-derived radiomic feature called gray mean, all of the identified radiomic features were significantly different between the mutant-type of EGFR and wild-type of EGFR subgroups. **(B)** Evaluation of the predictive value of individual identified radiomic features for EGFR mutational status by receiver operating characteristic (ROC) analysis. *indicates that the value of the area under the curve (AUC) was significantly greater than a random guess (AUC = 0.5). As presented, all of the identified individual radiomic features were capable of discriminating EGFR mutated cases from cases without EGFR mutation, except for a CT-based radiomic feature called gray mean. In general, the PET-derived individual radiomic feature outperformed the conventional PET parameters in distinguishing the mutant-type of EGFR and wild-type of EGFR subgroups. EGFR, epidermal growth factor receptor; PET, positron emission tomography; CT, computed tomography; EGFR+, mutant-type of EGFR; EGFR-, wild-type of EGFR.

As represented in the box plots in Figure 4A, marked differences existed between the EGFR+ subgroup and EGFR- subgroup in regard to all of the identified radiomic features included in the developed PET/CT radiomic signature [a total of 7 PET-derived radiomic features (concavity, gray mean, homogeneity, ASM, entropy, contrast, and correlation) and a CT-based radiomic feature called gray span] except for a CT-based radiomic feature called gray mean. The abilities of all of these identified radiomics features to predict EGFR mutational status in NSCLC were evaluated by assessing the AUC (Figure 4B). All of the identified PET-derived and CT-based radiomic features (gray span) were significantly predictive of EGFR mutational status (*P* < 0.05). The discriminative power ranged from AUC = 0.609 for concavity to AUC = 0.776 for homogeneity with respect to radiomic features based on PET images, and ranged from AUC = 0.590 for gray mean (*P* > 0.05) to AUC = 0.665 (*P* < 0.05) for gray span regarding to CT images-derived radiomic features. As clearly presented in Figure 4B, all of the PET radiomic features significantly outperformed the three significant conventional PET parameters (SUVmean, SUVmax, and SUVpeak).

## Discussion

Due to the high incidence and mortality associated with NSCLC, the early precise determination of some of the most common somatic mutations, such as EGFR mutational status, will be beneficial in improving lesion differentiation, responses to predictions and evaluations, and prognostication (7–9). A growing body of evidence has illustrated that radiomic assessments of the tumor imaging phenotype captured in integrated PET/CT images markedly facilitated tumor management, including differential diagnosis, tumor staging, response evaluation, and survival prediction (25, 34, 35, 38–40). Unfortunately, few studies have focused on evaluating the performance of radiomics derived from PET/CT in somatic mutation prediction for NSCLC patients (30, 31, 36).

In the present study, the aim was to reveal the association between PET/CT radiomic features with EGFR mutational status and evaluate their ability to predict mutational status in NSCLC. In general, radiomic signatures based on PET/CT images indicated a stronger predictive power for the EGFR mutation than the CT radiomic signature and conventional PET parameters. The results revealed that tumors with EGFR mutations tended to have a more irregular boundary (higher concavity), an overall lower randomness and complexity in a gray-level distribution (higher gray mean, higher ASM and lower entropy) and a higher heterogeneity (lower homogeneity, lower correlation, and higher contrast) in comparison with tumors without an EGFR mutation.

Consistent with the widely acceptable notion that tumors with a EGFR mutation are more indolent than tumors without a EGFR mutation (50), the metabolic parameter measurements in our study, such as SUVmax, SUVmean, and SUVpeak, were notably decreased in the tumors bearing EGFR mutations when in contrast with those observed in tumors without EGFR mutations. Results from Mak et al. and Na et al. also indicated a decreased SUVmax in NSCLC patients with a EGFR mutation compared with in those without a EGFR mutation (51, 52), which is in agreement with our results. Conversely, reports from Huang et al. and Ko et al. suggested that a higher SUVmax was more likely to predict the presence of an EGFR mutation (50, 53), whereas other previous results assumed no evident correlation between EGFR mutational status and PET metabolic parameters (54–56). The discrepancies in these aforementioned studies may be attributed to the patient demographics and ethnicity. Further analysis on larger cohorts and in different countries are needed to resolve this issue. The overall unsatisfactory performance of conventional CT and/or conventional PET features in the prediction of EGFR mutation status inspired us to develop superior radiomic indices to ascertain the somatic mutational status. Depending on the imaging modality, texture analysis of heterogeneity through radiomics conveys different meanings. PET-based radiomic analysis refers to the variability of the metabolic phenotype, while CT-based radiomic analysis manifests the distribution pattern of tissue density. Even with the presence of a larger sample size and an external validation design for previous studies with respect to genotype-phenotype interaction in NSCLC (30, 31, 36), these investigation merely performed CT-based radiomics (31, 36) or PET-based radiomics alone (26) for discrimination between patients with positive somatic mutations and those without somatic mutation. To determine this, we performed comprehensive radiomic analysis based on combined PET/CT images to evaluate their performances in EGFR mutation prediction in NSCLC. In contrast to previous reports which chose individual radiomic biomarkers in radiomic analysis in order to identify somatic mutations in NSCLC (30, 36), a radiomic signature that combined multiple radiomic features was established in the present study, as one single radiomic parameter is not sufficient to detect the gross heterogeneity in tumor lesions. The identification of a radiomic signature predictive of EGFR mutational status would be helpful in precision medicine for NSCLC. It was assumed that radiomic features based on PET and CT images were complementary to each other, and a radiomic signature based on combining PET and CT radiomic features could substantially improve its predictive power for EGFR mutational status. In our investigation, the developed PET/CT derived radiomic signature exhibited a comparable predictive value to that of the radiomic signature based on PET images alone in differentiating the mutant-type of EGFR and wild-type of EGFR subgroups, whereas the established radiomic signature based on PET images alone significantly outperformed the radiomic signature based on CT images alone. A further study involving a larger sample size and more extracted radiomic features is required to be able to ascertain the outperformance of PET/CT-derived radiomic signature over PET alone based radiomic signatures in EGFR mutation prediction in NSCLC.

Despite the valuable results described above, there are several limitations in the present study. First of all, owing to the retrospective nature of the study, the acquisition, reconstruction and delineation settings were not standardized or optimized for the patients included in this investigation. As reported previously, the repeatability of radiomics could be markedly influenced by all these parameters (33, 57–60). Secondly, partial volume effects as a result of the limited PET spatial resolution may lead to an underestimation of metabolic measurements in PET images (61), and probably affect the PET-based radiomics for NSCLC patients with relatively smaller tumor volumes. Furthermore, the lack of respiratory-gated PET/CT imaging (62) may induce image blurring, which consequently led to a relatively poor performance in the quantification of the imaging phenotype. In the end, due to the small sample size of this study, we did not perform a robust external validation by applying a strict statistical design with independent training and validation cohorts in a large number of patients.

## Conclusions

Tumor imaging phenotypes that are driven by somatic mutations may be quantitatively measured by radiomic features extracted from PET/CT images for NSCLC patients. Radiomic features outperformed conventional PET parameters in the prediction of EGFR mutational status. PET/CT radiomic signatures combined with clinical factors exhibited a further improved performance. More importantly, in future investigations, we should be aware that intra-tumor heterogeneity is a big challenge for imaging and genetic correlation study. This is hard to overcome in current radiogenomic study design. The recently proposed habitat imaging study may have the potential to shed light on this issue (63, 64).

## Data Availability Statement

All datasets generated for this study are included in the manuscript/Supplementary Files.

## Ethics Statement

The studies involving human participants were reviewed and approved by Tianjin Medical University Cancer Hospital Institutional Ethics Committee. The patients/participants provided their written informed consent to participate in this study.

## Author Contributions

WM, WX, and XL conceived and designed the study. DD, JL, PC, LZ, and GY performed the retrospective study. GY, WM, YZ, and JL interpreted the data. XL, WM, and YZ wrote the paper. WM and WX supervised the study, reviewed, and edited the manuscript. All authors approved the final manuscript.

### Conflict of Interest

The authors declare that the research was conducted in the absence of any commercial or financial relationships that could be construed as a potential conflict of interest.

## References

[1] Siegel RL Miller KD Jemal A Cancer Statistics, 2017. CA Cancer J Clin. (2017) 67:7–30. 10.3322/caac.21387 28055103

[2] Goldstraw P Ball D Jett JR Le Chevalier T Lim E Nicholson AG. Non-small-cell lung cancer. Lancet. (2011) 378:1727–40. 10.1016/S0140-6736(10)62101-0 21565398

[3] De Rosa V Iommelli F Monti M Fonti R Votta G Stoppelli MP. Reversal of Warburg effect and reactivation of oxidative phosphorylation by differential inhibition of EGFR signaling pathways in non-small cell lung cancer. Clin Cancer Res. (2015) 21:5110–20. 10.1158/1078-0432.CCR-15-0375 26216352

[4] Steuer CE Ramalingam SS. Targeting EGFR in lung cancer: lessons learned and future perspectives. Mol Aspects Med. (2015) 45:67–73. 10.1016/j.mam.2015.05.004 26022942 PMC5024712

[5] Tricker EM Xu C Uddin S Capelletti M Ercan D Ogino A. Combined EGFR/MEK inhibition prevents the emergence of resistance in EGFR-mutant lung cancer. Cancer Discov. (2015) 5:960–71. 10.1158/2159-8290.CD-15-0063 26036643 PMC4824006

[6] Jänne PA Yang JC Kim DW Planchard D Ohe Y Ramalingam SS. AZD9291 in EGFR inhibitor-resistant non-small-cell lung cancer. N Engl J Med. (2015) 372:1689–99. 10.1056/NEJMoa1411817 25923549

[7] Kobayashi S Boggon TJ Dayaram T Jänne PA Kocher O Meyerson M. EGFR mutation and resistance of non-small-cell lung cancer to gefitinib. N Engl J Med. (2005) 352:786–92. 10.1056/NEJMoa044238 15728811

[8] Heuckmann JM Rauh D Thomas RK. Epidermal growth factor receptor (EGFR) signaling and covalent EGFR inhibition in lung cancer. J Clin Oncol. (2012) 30:3417–20. 10.1200/JCO.2012.43.1825 22915655

[9] Paez JG Jänne PA Lee JC Tracy S Greulich H Gabriel S. EGFR mutations in lung cancer: correlation with clinical response to gefitinib therapy. Science. (2004) 304:1497–500. 10.1126/science.1099314 15118125

[10] Ellison G Zhu G Moulis A Dearden S Speake G McCormack R. EGFR mutation testing in lung cancer: a review of available methods and their use for analysis of tumour tissue and cytology samples. J Clin Pathol. (2013) 66:79–89. 10.1136/jclinpath-2012-201194 23172555 PMC3582044

[11] Iwama E Sakai K Azuma K Harada D Nosaki K Hotta K. Exploration of resistance mechanisms for epidermal growth factor receptor-tyrosine kinase inhibitors based on plasma analysis by digital polymerase chain reaction and next-generation sequencing. Cancer Sci. (2018) 109:3921–33. 10.1111/cas.13820 30289575 PMC6272092

[12] Grootjans W de Geus-Oei LF Troost EG Visser EP Oyen WJ Bussink J. PET in the management of locally advanced and metastatic NSCLC. Nat Rev Clin Oncol. (2015) 12:395–407. 10.1038/nrclinonc.2015.75 25917254

[13] Meijer TWH de Geus-Oei LF Visser EP Oyen WJG Looijen-Salamon MG Visvikis D Tumor delineation and quantitative assessment of glucose metabolic rate within histologic subtypes of non-small cell lung cancer by using dynamic (18)F fluorodeoxyglucose PET. Radiology. (2017) 283:547–59. 10.1148/radiol.2016160329 27846378

[14] Lim SO Li CW Xia W Lee HH Chang SS Shen J. EGFR signaling enhances aerobic glycolysis in triple-negative breast cancer cells to promote tumor growth and immune escape. Cancer Res. (2016) 76:1284–96. 10.1158/0008-5472.CAN-15-2478 26759242 PMC4775355

[15] Cho A Hur J Moon YW Hong SR Suh YJ Kim YJ. Correlation between EGFR gene mutation, cytologic tumor markers, 18F-FDG uptake in non-small cell lung cancer. BMC Cancer. (2016) 16:224. 10.1186/s12885-016-2251-z 26979333 PMC4793740

[16] Benz MR Herrmann K Walter F Garon EB Reckamp KL Figlin R. (18)F-FDG PET/CT for monitoring treatment responses to the epidermal growth factor receptor inhibitor erlotinib. J Nucl Med. (2011) 52:1684–9. 10.2967/jnumed.111.095257 22045706 PMC5021512

[17] Takahashi R Hirata H Tachibana I Shimosegawa E Inoue A Nagatomo I. Early [18F]Fluorodeoxyglucose positron emission tomography at two days of gefitinib treatment predicts clinical outcome in patients with adenocarcinoma of the lung. Clin Cancer Res. (2012) 18:220–8. 10.1158/1078-0432.CCR-11-0868 22019513

[18] van Gool MH Aukema TS Sinaasappel M Valdés Olmos RA Klomp HM. Tumor heterogeneity on (18)F-FDG-PET/CT for response monitoring in non-small cell lung cancer treated with erlotinib. J Thorac Dis. (2016) 8:E200–3. 10.21037/jtd.2016.02.10 27076970 PMC4805797

[19] Aukema TS Kappers I Olmos RA Codrington HE van Tinteren H van Pel R. Is 18F-FDG PET/CT useful for the early prediction of histopathologic response to neoadjuvant erlotinib in patients with non-small cell lung cancer? J Nucl Med. (2010) 51:1344–8. 10.2967/jnumed.110.076224 20720059

[20] Ho TY Chou PC Yang CT Tsang NM Yen TC. Total lesion glycolysis determined per RECIST 1.1 criteria predicts survival in EGFR mutation-negative patients with advanced lung adenocarcinoma. Clin Nucl Med. (2015) 40:e295–9. 10.1097/RLU.0000000000000774 25783515

[21] Keam B Lee SJ Kim TM Paeng JC Lee SH Kim DW. Total lesion glycolysis in positron emission tomography can predict Gefitinib outcomes in non-small-cell lung cancer with activating EGFR mutation. J Thorac Oncol. (2015) 10:1189–94. 10.1097/JTO.0000000000000569 26200273

[22] Hatt M Tixier F Visvikis D Cheze Le Rest C. Radiomics in PET/CT: more than meets the eye? J Nucl Med. (2017) 58:365–6. 10.2967/jnumed.116.184655 27811126

[23] Chicklore S Goh V Siddique M Roy A Marsden PK Cook GJ. Quantifying tumour heterogeneity in 18F-FDG PET/CT imaging by texture analysis. Eur J Nucl Med Mol Imaging. (2013) 40:133–40. 10.1007/s00259-012-2247-0 23064544

[24] Wu J Aguilera T Shultz D Gudur M Rubin DL Loo BW. Early-stage non-small cell lung cancer: quantitative imaging characteristics of (18)F Fluorodeoxyglucose PET/CT allow prediction of distant metastasis. Radiology. (2016) 281:270–8. 10.1148/radiol.2016151829 27046074 PMC5047129

[25] Wu J Tha KK Xing L Li R. Radiomics and radiogenomics for precision radiotherapy. J Radiat Res. (2018) 59:i25–31. 10.1093/jrr/rrx102 29385618 PMC5868194

[26] Gan Y Shi C Inge L Hibner M Balducci J Huang Y. Differential roles of ERK and Akt pathways in regulation of EGFR-mediated signaling and motility in prostate cancer cells. Oncogene. (2010) 29:4947–58. 10.1038/onc.2010.240 20562913

[27] Elstrom RL Bauer DE Buzzai M Karnauskas R Harris MH Plas DR. Akt stimulates aerobic glycolysis in cancer cells. Cancer Res. (2004) 64:3892–9. 10.1158/0008-5472.CAN-03-2904 15172999

[28] Yun J Rago C Cheong I Pagliarini R Angenendt P Rajagopalan H. Glucose deprivation contributes to the development of KRAS pathway mutations in tumor cells. Science. (2009) 325:1555–9. 10.1126/science.1174229 19661383 PMC2820374

[29] Ying H Kimmelman AC Lyssiotis CA Hua S Chu GC Fletcher-Sananikone E. Oncogenic Kras maintains pancreatic tumors through regulation of anabolic glucose metabolism. Cell. (2012) 149:656–70. 10.1016/j.cell.2012.01.058 22541435 PMC3472002

[30] Yip SS Kim J Coroller TP Parmar C Velazquez ER Huynh E. Associations between somatic mutations and metabolic imaging phenotypes in non-small cell lung cancer. J Nucl Med. (2017) 58:569–76. 10.2967/jnumed.116.181826 27688480 PMC5373502

[31] Rios Velazquez E Parmar C Liu Y Coroller TP Cruz G Stringfield O. Somatic mutations drive distinct imaging phenotypes in lung cancer. Cancer Res. (2017) 77:3922–30. 10.1158/0008-5472.CAN-17-0122 28566328 PMC5528160

[32] Wu J Li X Teng X Rubin DL Napel S Daniel BL. Magnetic resonance imaging and molecular features associated with tumor-infiltrating lymphocytes in breast cancer. Breast Cancer Res. (2018) 20:101. 10.1186/s13058-018-1039-2 30176944 PMC6122724

[33] Wu J Sun X Wang J Cui Y Kato F Shirato H. Identifying relations between imaging phenotypes and molecular subtypes of breast cancer: model discovery and external validation. J Magn Reson Imaging. (2017) 46:1017–27. 10.1002/jmri.25661 28177554 PMC5548657

[34] Thawani R McLane M Beig N Ghose S Prasanna P Velcheti V. Radiomics and radiogenomics in lung cancer: a review for the clinician. Lung Cancer. (2018) 115:34–41. 10.1016/j.lungcan.2017.10.015 29290259

[35] Lee G Park H Sohn I Lee SH Song SH Kim H. Comprehensive computed tomography radiomics analysis of lung adenocarcinoma for prognostication. Oncologist. (2018) 23:806–13. 10.1634/theoncologist.2017-0538 29622699 PMC6058328

[36] Liu Y Kim J Balagurunathan Y Li Q Garcia AL Stringfield O. Radiomic features are associated with EGFR mutation status in lung adenocarcinomas. Clin Lung Cancer. (2016) 17:441–8.e6. 10.1016/j.cllc.2016.02.001 27017476 PMC5548419

[37] Ortiz-Ramon R Larroza A Arana E Moratal D. A radiomics evaluation of 2D and 3D MRI texture features to classify brain metastases from lung cancer and melanoma. Conf Proc IEEE Eng Med Biol Soc. (2017) 2017:493–6. 10.1109/EMBC.2017.8036869 29059917

[38] Kirienko M Cozzi L Rossi A Voulaz E Antunovic L Fogliata A. Ability of FDG PET and CT radiomics features to differentiate between primary and metastatic lung lesions. Eur J Nucl Med Mol Imaging. (2018) 45:1649–60. 10.1007/s00259-018-3987-2 29623375

[39] Takeda K Takanami K Shirata Y Yamamoto T Takahashi N Ito K. Clinical utility of texture analysis of 18F-FDG PET/CT in patients with Stage I lung cancer treated with stereotactic body radiotherapy. J Radiat Res. (2017) 58:862–9. 10.1093/jrr/rrx050 29036692 PMC5710655

[40] Oikonomou A Khalvati F Tyrrell PN Haider MA Tarique U Jimenez-Juan L. Radiomics analysis at PET/CT contributes to prognosis of recurrence and survival in lung cancer treated with stereotactic body radiotherapy. Sci Rep. (2018) 8:4003. 10.1038/s41598-018-22357-y 29507399 PMC5838232

[41] Ma W Zhao Y Ji Y Guo X Jian X Liu P. Breast cancer molecular subtype prediction by mammographic radiomic features. Acad Radiol. (2019) 26:196–201. 10.1016/j.acra.2018.01.023 29526548 PMC8082943

[42] Ma W Ji Y Qi L Guo X Jian X Liu P. Breast cancer Ki67 expression prediction by DCE-MRI radiomics features. Clin Radiol. (2018) 73:909.e1–5. 10.1016/j.crad.2018.05.027 29970244

[43] Rodenacker K Bengtsson E. A feature set for cytometry on digitized microscopic images. Anal Cell Pathol. (2003) 25:1–36. 10.1155/2003/548678 12590175 PMC4618906

[44] Aerts HJ Velazquez ER Leijenaar RT Parmar C Grossmann P Carvalho S. Decoding tumour phenotype by noninvasive imaging using a quantitative radiomics approach. Nat Commun. (2014) 5:4006. 10.1038/ncomms5644 24892406 PMC4059926

[45] Li Z Mao Y Huang W Li H Zhu J Li W. Texture-based classification of different single liver lesion based on SPAIR T2W MRI images. BMC Med Imaging. (2017) 17:42. 10.1186/s12880-017-0212-x 28705145 PMC5508617

[46] Tamura H Mori S Yamawaki T Textural features corresponding to visual perception. IEEE Trans Syst Man Cybern. (1978) 8:460–73. 10.1109/TSMC.1978.4309999

[47] Tibshirani R. The lasso method for variable selection in the Cox model. Stat Med. (1997) 16:385–95. 10.1002/(SICI)1097-0258(19970228)16:4<385::AID-SIM380>3.0.CO;2-3 9044528

[48] Benjamini Y Hochberg Y Controlling the false discovery rate: a practical and powerful approach to multiple testing. J R Stat Soc Series B Stat Methodol. (1995) 57:289–300. 10.1111/j.2517-6161.1995.tb02031.x

[49] Jänne PA Johnson BE. Effect of epidermal growth factor receptor tyrosine kinase domain mutations on the outcome of patients with non-small cell lung cancer treated with epidermal growth factor receptor tyrosine kinase inhibitors. Clin Cancer Res. (2006) 12:4416s−20. 10.1158/1078-0432.CCR-06-0555 16857820

[50] Ko KH Hsu HH Huang TW Gao HW Shen DH Chang WC Value of (1)(8)F-FDG uptake on PET/CT and CEA level to predict epidermal growth factor receptor mutations in pulmonary adenocarcinoma. Eur J Nucl Med Mol Imaging. (2014) 41:1889–97. 10.1007/s00259-014-2802-y 24852187

[51] Mak RH Digumarthy SR Muzikansky A Engelman JA Shepard JA Choi NC. Role of 18F-fluorodeoxyglucose positron emission tomography in predicting epidermal growth factor receptor mutations in non-small cell lung cancer. Oncologist. (2011) 16:319–26. 10.1634/theoncologist.2010-0300 21339258 PMC3228101

[52] Na II Byun BH Kim KM Cheon GJ Choe du H Koh JS. 18F-FDG uptake and EGFR mutations in patients with non-small cell lung cancer: a single-institution retrospective analysis. Lung Cancer. (2010) 67:76–80. 10.1016/j.lungcan.2009.03.010 19371962

[53] Huang CT Yen RF Cheng MF Hsu YC Wei PF Tsai YJ. Correlation of F-18 fluorodeoxyglucose-positron emission tomography maximal standardized uptake value and EGFR mutations in advanced lung adenocarcinoma. Med Oncol. (2010) 27:9–15. 10.1007/s12032-008-9160-1 19130320

[54] Choi YJ Cho BC Jeong YH Seo HJ Kim HJ Cho A. Correlation between (18)f-fluorodeoxyglucose uptake and epidermal growth factor receptor mutations in advanced lung cancer. Nucl Med Mol Imaging. (2012) 46:169–75. 10.1007/s13139-012-0142-z 24900056 PMC4043041

[55] Putora PM Fruh M Muller J FDG-PET SUV-max values do not correlate with epidermal growth factor receptor mutation status in lung adenocarcinoma. Respirology. (2013) 18:734–5. 10.1111/resp.12083 23489365

[56] Liu A Han A Zhu H Ma L Huang Y Li M The role of metabolic tumor volume (MTV) measured by [18F] FDG PET/CT in predicting EGFR gene mutation status in non-small cell lung cancer. Oncotarget. (2017) 8:33736–44. 10.18632/oncotarget.16806 28422710 PMC5464907

[57] van Velden FH Kramer GM Frings V Nissen IA Mulder ER de Langen AJ. Repeatability of radiomic features in non-small-cell lung cancer [(18)F]FDG-PET/CT studies: impact of reconstruction and delineation. Mol Imaging Biol. (2016) 18:788–95. 10.1007/s11307-016-0940-2 26920355 PMC5010602

[58] Sollini M Cozzi L Antunovic L Chiti A Kirienko M. PET Radiomics in NSCLC: state of the art and a proposal for harmonization of methodology. Sci Rep. (2017) 7:358. 10.1038/s41598-017-00426-y 28336974 PMC5428425

[59] Desseroit MC Tixier F Weber WA Siegel BA Cheze Le Rest C Visvikis D. Reliability of PET/CT shape and heterogeneity features in functional and morphologic components of non-small cell lung cancer tumors: a repeatability analysis in a prospective multicenter cohort. J Nucl Med. (2017) 58:406–11. 10.2967/jnumed.116.180919 27765856 PMC5331937

[60] Shiri I Rahmim A Ghaffarian P Geramifar P Abdollahi H Bitarafan-Rajabi A. The impact of image reconstruction settings on 18F-FDG PET radiomic features: multi-scanner phantom and patient studies. Eur Radiol. (2017) 27:4498–509. 10.1007/s00330-017-4859-z 28567548

[61] Le Pogam A Hatt M Descourt P Boussion N Tsoumpas C Turkheimer FE. Evaluation of a 3D local multiresolution algorithm for the correction of partial volume effects in positron emission tomography. Med Phys. (2011) 38:4920–3. 10.1118/1.3608907 21978037 PMC3485215

[62] Wellman TJ Winkler T Costa EL Musch G Harris RS Venegas JG. Measurement of regional specific lung volume change using respiratory-gated PET of inhaled 13N-nitrogen. J Nucl Med. (2010) 51:646–53. 10.2967/jnumed.109.067926 20237036 PMC3177560

[63] Wu J Cao G Sun X Lee J Rubin DL Napel S. Intratumoral spatial heterogeneity at perfusion MR imaging predicts recurrence-free survival in locally advanced breast cancer treated with neoadjuvant chemotherapy. Radiology. (2018) 288:26–35. 10.1148/radiol.2018172462 29714680 PMC6029132

[64] Wu J Gong G Cui Y Li R. Intratumor partitioning and texture analysis of dynamic contrast-enhanced (DCE)-MRI identifies relevant tumor subregions to predict pathological response of breast cancer to neoadjuvant chemotherapy. J Magn Reson Imaging. (2016) 44:1107–15. 10.1002/jmri.25279 27080586 PMC5061585

