# Supplementary material for: Predictive Power of a Radiomic Signature Based on 18F-FDG PET/CT Images for EGFR Mutational Status in NSCLC
Source: Front Oncol. 2019 Oct 15;9:1062. doi: 10.3389/fonc.2019.01062 (PMC6803612; doi:10.3389/fonc.2019.01062)
Supplement: Supplementary file 1 [file Data_Sheet_1.docx]

**Supplemental Document S1**

Formula used to calculate Greyscale statistic (GSS) features, Gray-Level Co-Occurrence Matrix based (GLCM) features, Gray level-gradient co-occurrence matrix (GGCM) features and Gray-level difference statistics (GLDS) features in our work.

***Greyscale statistic (GSS) features:***

Greyscale statistic features calculated from the histogram of the tumor voxel intensities, Let X denote the three dimensional image matrix with N voxels. Their mathematical expressions are as below:

1. Mean

$$M_{1}=\frac{1}{N}\sum_{\text{i}}^{N} X(i)$$

1. Standard Deviation

Where $\bar{X}$is the mean of X .

1. Skewness

1. Kurtosis

1. The Fifth Center Moment

1. The Sixth Center Moment

1. Span

The maximum minus the minimum intensity value of X .

***Gray-Level Co-Occurrence Matrix based (GLCM) features:***

A GLCM is defined as $P(i,j;\delta,\alpha)$, a matrix with size $N_{\text{g}}\times N_{\text{g}}$ describing the secondorder joint probability function of an image, where the $\left(i,j\right)$th element represents the number of times the combination of intensity levels $\text{i}$and $\text{j}$ occur in two pixels in the image, that are separated by a distance of $\delta$ pixels in direction $\alpha$ , and $N_{g}$is the number of discrete gray level intensities. In this artical, prior to the computation of texture features, the full intensity range of the tumour region was quantized to a smaller number of gray levels 16. For angles = 0^0^, 45^0^, 90^0^, and 135^0^, we computed four values for each of the above texture measures. Each feature was computed using a distance of one pixel.

**Let:**

$P(i,j)$ be the co-occurrence matrix for an arbitrary $\delta$and $\alpha$,

$\mu$be the mean of $P(i,j)$,

$\mu_{\text{x}}$be the moean of $\text{p}_{x}$,

$\mu_{\text{y}}$be the moean of $\text{p}_{\text{y}}$,

$\sigma_{\text{x}}$be the moean of $\sigma_{\text{x}}$,

$\sigma_{\text{y}}$be the moean of $\sigma_{\text{y}}$.

Their mathematical expressions are as below:

1. Angular Second Moment (ASM),

1. Contrast,

1. Correlation,

$$F_{3}=\left(\sum_{i=1}^{N_{g}} \sum_{j=1}^{N_{g}} \text{i}jP(i,j)-\mu_{i}(i)\mu_{j}(j)\right)/\sigma_{x}(i)\sigma_{x}(j)$$

1. Inverse difference moment (IDM)

1. Entropy

1. Sum of Squares

$$F_{6}=\sum_{i=1}^{N_{g}} \sum_{j=1}^{N_{g}} (i-\mu)^{2}P(i,j)$$

1. Homogeneity.

1. Autocorrelation

$$F_{8}=\sum_{i=1}^{N_{g}} \sum_{j=1}^{N_{g}} \text{i}jP(i,j)$$

***Gray level-gradient co-occurrence matrix (GGCM) features:***

The element $h(i,j)$ of GLCM is defined as the probability of the pixel number which has gray value *i* in the normalized gray image *F*(*m,n*) and gradient value *j* in the normalized gradient image

*G*(*m,n*).Then, count the number of pixels which satisfy *F*(*m,n*)=*i* and *G*(*m,n*)=*j*, take it as H_ij_. The
total amount of H_ij_ were calculated by

$H=\text{、}\sum_{i=1}^{Ng} \sum_{j=1}^{Ng} H_{ij}$.

Then, the normalized GLGCM $\hat{H}_{\text{ij}}$can be caclulated by formula:

$$H=\text{、}H_{ij}/\left(N_{g}\times N_{s}\right)$$

where, i=1,2,...N_g_, j=1,2,..N_s_,, N_g_=32. The feature computing formula is described as follow.

1. Small Grads Dominance

$$T_{1}=\text{、}\left[\sum_{i=1}^{Ng} \sum_{j=1}^{Ng} \frac{H_{ij}}{\text{j}^{2}}\right]/H$$

1. Big Grads Dominance

$$T_{2}=\text{、}\left[\sum_{i=1}^{Ng} \sum_{j=1}^{Ng} \text{j}^{2}H_{ij}\right]/H$$

1. Gray Asymmetry

$$T_{3}=\text{、}\sum_{\text{i}=1}^{Ng} \left[\sum_{j=1}^{Ns} H_{ij}\right]^{2}/H$$

1. Grads Asymmetry

$$T_{4}=\text{、}\sum_{\text{j}=1}^{Ng} \left[\sum_{i=1}^{Ns} H_{ij}\right]^{2}/H$$

1. Energy

1. Gray Mean

1. Grads Mean

1. Gray Variance

1. Grads Variance

1. Correlation

1. Gray Entropy

1. Grads Entropy

1. Entropy

1. Inertia

$$T_{14}=\text{、}-\sum_{i=1}^{Ng} \sum_{j=1}^{N\text{s}} \text{（}\left(i-j\right)^{2}\hat{H}_{\text{ij}}$$

1. Homogeneity

$$T_{15}=\text{、}-\sum_{i=1}^{Ng} \sum_{j=1}^{N\text{s}} \text{（}\frac{1}{1+\left(i-j\right)^{2}}\hat{H}_{\text{ij}}$$

***Gray-level difference statistics (GLDS) features***

Gray-level difference statistics is commonly used in texture analysis. Suppose $\text{g}(x,y)$is a pixel point of image, the gray difference between this pixel and its neighborhood pixel $\text{g}(x+\Delta\text{x},y+\Delta y)$is as below:

$$\text{g}_{\Delta}\text{(x,y)}=\text{g(x,y)-g}(x+\Delta\text{x},y+\Delta y)$$

$\text{g}_{\Delta}(x,y)$ in (1) is defined as gray difference.$\text{g}_{\Delta}(x,y)$of each pixel is caculated by moving $(x,y)$ throught the image. Suppose gray difference level is m , the number of each $\text{g}_{\Delta}(x,y)$ is counted up,and histogram of $\text{g}_{\Delta}(x,y)$ is drawn. Finally occurred probability $\text{P}_{\Delta}(\text{i})$of each $\text{g}_{\Delta}(x,y)$ is caculated based on histogram. Texture characteristic is closely related to . If $\text{P}_{\Delta}(\text{i})$ changes rapidly along with variation of i, texture of image is coarse, whereas it is fine if $\text{P}_{\Delta}(\text{i})$ distributes smoothly. In this study , texture features including contrast,angular second moment, mean and entropy were extracted from the gray level difference statistics. Their mathematical expressions are as below:

1. Contrast

1. Angular Second Moment

1. Mean

1. Entropy
